# Paediatric neurosurgical implications of a ribosomopathy: illustrative case and literature review

**DOI:** 10.1007/s00381-021-05208-6

**Published:** 2021-05-21

**Authors:** Suzanne Murphy, Gabriella Grima, Kshitij Mankad, Kristian Aquilina

**Affiliations:** 1 School of Medicine, Royal College of Surgeons, Dublin, Ireland; 2 Department of Neurosurgery, Great Ormond Street Hospital NHS Foundation Trust, London, UK; 3 Department of Neuroradiology, Great Ormond Street Hospital NHS Foundation Trust, London, UK

**Keywords:** Ribosomopathy, Labrune syndrome, SNORD118, LCC, Hydrocephalus

## Abstract

Ribosomopathies are rare, recently defined entities. One of these, Labrune syndrome, is recognisable radiologically by its distinctive triad of leukoencephalopathy, intracranial calcifications and cysts (LCC). These cysts may have neurosurgical implications at different ages because of their progressive expansion and local mass effect. The aetiology of LCC is related to a widespread cerebral microangiopathy and is due to a genetic mutation in SNORD118, responsible for stabilisation of the large ribosomal subunit during assembly.

## Introduction

Ribosomopathies are a diverse group of tissue-specific diseases caused by reduced expression, or mutation, of factors necessary for the synthesis of ribosomes [1]. These include, among others, Treacher Collins syndrome, Diamond Blackfan and some types of congenital cirrhosis. Leukoencephalopathy with cerebral calcifications and cysts (LCC), also known as Labrune syndrome, was first described in three children in 1996, who presented with cognitive deterioration and seizures beginning in early infancy [2, 3]. Several cases have now been reported in adults and children [4–30].

The triad of LCC is evident on imaging. The primary cause is an isolated cerebrovascular microangiopathy, inherited in an autosomal recessive manner, and related to a mutation in the SNORD118 gene that is essential for maturation of the 60S subunit of the ribosome. The presence of multiple cysts, often deep within the brain in eloquent locations, and their tendency to recur, represents a significant neurosurgical challenge. The objective of this study is to illustrate a new paediatric case of LCC and review the neurosurgical strategies that have been used to manage this rare and difficult condition.

## Case illustration

**Fig. 1:**
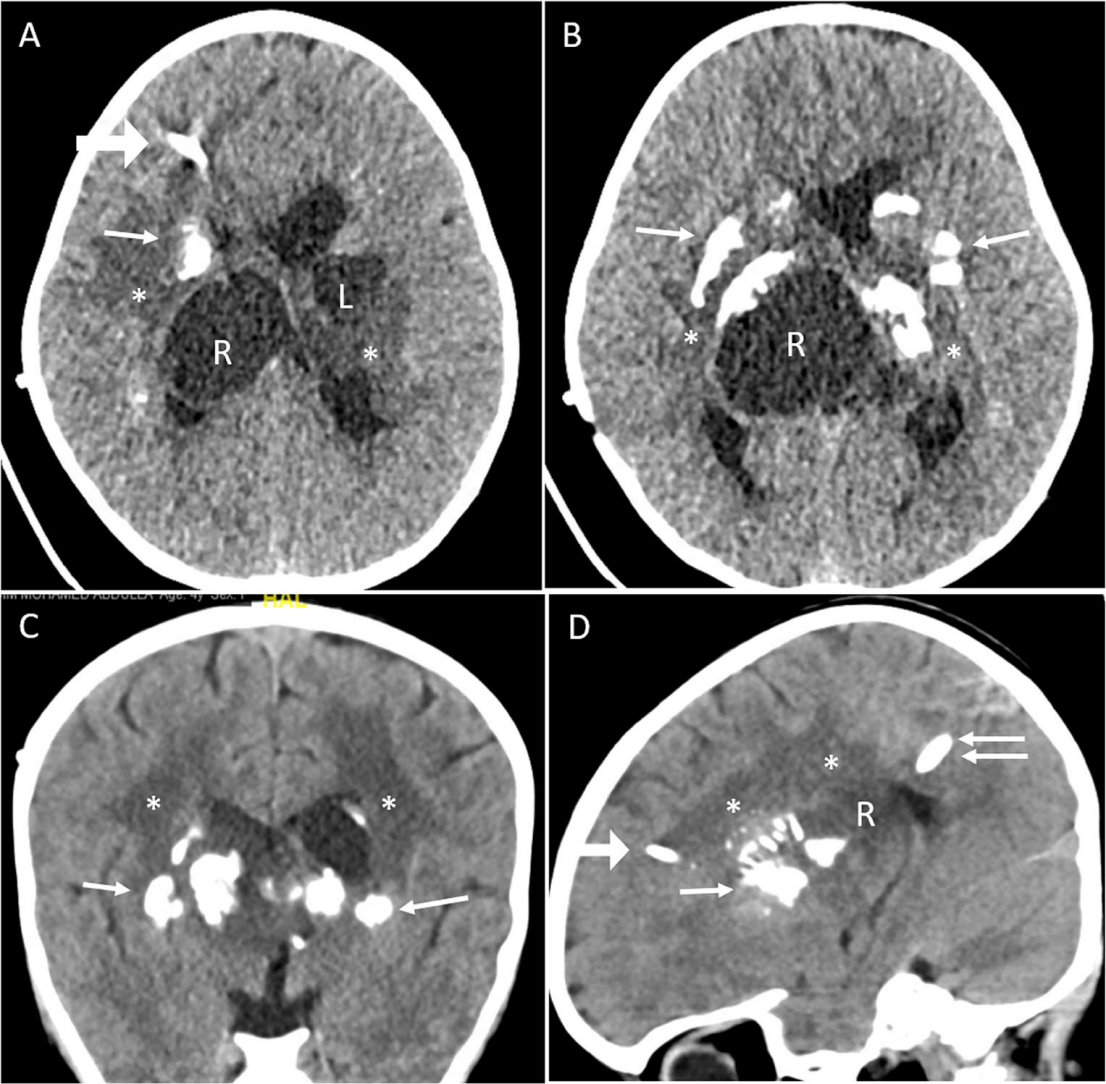
Computed tomography scans, axial (**A**, **B**), coronal (**C**), and sagittal (**D**), to demonstrate the triad of calcification (single arrows), thalamic cysts on the left (L) and right (R), and leukoencephalopathy (*) in the patient illustrated. The arrowhead in (**A**) and (**D**) denotes the ventricular catheter of the right ventriculoperitoneal shunt. The double arrow in (**D**) denotes the catheter directed to the now drained right thalamic cyst (R); this was attached to an Ommaya reservoir to allow repeated dra

A 4-year-old girl initially presented to another institution with a 1-week history of headache and vomiting. A CT scan of the brain showed bilateral thalamic cysts with calcifications. In order to exclude a thalamic tumour, she underwent stereotactic biopsy with drainage of the large left-sided cyst at the same time. She subsequently developed symptomatic hydrocephalus, and a right frontal VP shunt was inserted (Fig. 1A). The biopsy showed no evidence of tumour, but demonstrated areas of Rosenthal fibre deposition, calcification and demyelination. These findings on histology, together with the absence of tumour and the typical triad on imaging, confirmed the diagnosis of LCC.

She was referred to our institution 6 months later, with worsening headache, ataxia and bilateral coarse hand tremor. There was no limb weakness or visual disturbance. Imaging showed progressive enlargement of the right thalamic cyst, with mass effect on the midbrain and pons (Fig. 1B). Further drainage of the cyst was performed. A catheter and Ommaya reservoir were inserted under image guidance into the cyst and 60 ml of xanthochromic fluid was aspirated (Fig. 1). No cells or organisms were identified in the fluid. As an MRI scan would have required an additional general anaesthetic, a post-operative CT scan was carried out which demonstrated satisfactory drainage of the large right thalamic cyst (Fig. 1 C and D). The child then returned to her home country. No further aspirations were necessary over a 1-year follow-up period.

## Discussion

LCC is a rare progressive neurological disorder that can present at any age. We identified 39 cases reported in the last ten years (Table 1). Many adult patients reported symptoms from childhood [8, 9, 12, 20, 26]. LCC is clinically defined by the radiological triad, with development of asymmetric cysts, usually involving the thalami, basal ganglia, dentate nuclei and deep white matter, as well as diffuse hemispheric leukoencephalopathy and dystrophic calcification. Cerebral atrophy is absent. The primary change is the presence of microangiopathy in the brain, with ectasia of small cerebral blood vessels. There is deposition of Rosenthal fibres, with demyelination and calcification. Inflammation is minimal. Histology of the cyst wall shows no evidence of infective or neoplastic changes, with the MIB labelling index reported to be zero [18]. It is likely that the angiomatous changes and areas of focal thrombosis lead to microhaemorrhages and ischaemic necrosis, which coalesce into cysts or develop into areas of calcification. Loss of blood brain barrier integrity leads to white matter oedema. Inheritance is autosomal recessive, without X linkage [3, 19]. A germline biallelic mutation in SNORD 118, on chromosome 17p, encoding the small nucleolar RNA U8 (snoRNA U8), has been consistently identified in several cases [3]. There is a large variation in causative mutations, each causing variable loss of function. It is unclear why a ribosomal defect, which would be expected to cause a global disturbance, only leads to specific pathology restricted to the brain. It is likely that LCC occurs in the context of one null mutation and a paired milder mutation; the presence of two null mutations is lethal at the embryonic stage (Ref to Jenkinson 2016).

**Table 1:** Cases reported in the last ten years

Author and year	Age	Gender	Presenting complaint	Duration/onset of symptoms	Neuroimaging	Genetic testing obtained?	Treatment	Duration of follow-up	Outcomes
Fay et al. 2017 [8]	18	Male	Seizures, bradykinesia	Since 11 weeks of age	MRI (at age 6 years) showed bilateral cysts. Repeat MRI at 18 years showed increased calcifications.	Heterozygous for 2 variants in SNORD118: n.*5C>G and n.81G>A	Biweekly infusions of VEGF inhibitor bevacizumab for 1 year	1 year	At 1 year: Improvement in bradykinesia. MRI showed reduction in cyst volume and white matter lesions. Improvement appeared to plateau at 6 months.
Gupta et al. 2019 [10]	18	Female	Progressive left sided weakness, seizures and facial pain	8 months	CT—large cystic lesion	No	N/A	N/A	N/A
Iwama et al. 2017 [11]	61	Female	Seizures, headaches, depression	Seizures began at age 4	CT (at age 37) cerebral calcifications. CT (age 50): intracranial calcifications and ventricular leukoencephalopathy but no cysts. MRI and CT (age 61) occipital lobe haemorrhage, progressive ventricular dilatation and expanding leukoencephalopathy	Yes; heteromutation of c.38C>G and c.116G > C on different alleles	N/A	N/A	Progressive neurological decline
Iwasaki et al. 2017 [12]	11 months	Female	Complex febrile seizures	N/A	MRI: Brain calcifications, leukoencephalopathy and intracranial cyst	n.[39G>C]; [103G>A]	N/A	N/A	N/A
Iwasaki et al. 2017 [12]	1 month	Male	Epilepsy	N/A	MRI: Brain calcifications, leukoencephalopathy and intracranial cyst	n. [39G>C]; [72A>G]	N/A	N/A	N/A
Iwasaki et al. 2017 [12]	9 years 2 months	Male	Spastic hemiplegia and dystonia	N/A	MRI: brain calcifications and leukoencephalopathy	n. [3C>T]; [24C>T}	N/a	N/A	N/A
McNeill et al. 2017 [16]	6	Male	Progressive encephalopathy—intractable seizures, dystonia, chorea, spasticity and impaired cognition	N/A	MRI; diffuse white matter signal abnormalities and numerous calcifications throughout the brain in grey matter nuclei and juxtacortical U-fibres, periventricular white matter, brainstem, dentate nucleus of cerebellum and subcortical white matter.	Yes—heterozygous mutations in EARS2 c.328G>A (p.G110S) and C.1045G>A (p.E349K)	N/A	10 years	Patient died at age 16. Autopsy showed vasculopathy throughout the CNS with secondary ischaemic lesions and mineralisation.
Osman et al. 2019 [19]	30	Female	Seizures	19 years	MRI: Bilateral and symmetric white-matter lesions, multiple calcifications and cysts	Compound heterozygous mutations n.72A>G and n.92C>T	Numerous surgical procedures	N/A	Progressive deterioration. Died aged 30 after fall from wheelchair.
Pahuja, et al. 2017 [20]	10	Male	Headache, vomiting and focal seizures	1 year	CT: Luckenschadel skull with intracranial calcifications. MRI: cystic changes and hydrocephalus	No	Family refused surgical intervention. Commenced antiepileptic drugs.	N/A	AEDs helped relieve symptoms
Shtaya et al. 2019 [23]	12	Male	Worsening headache	3 months (Hx of developmental delay	MRI; widespread calcifications, large left cerebellar cyst	Yes—n.72A>G and n.*roG>T biallelic variants in SNORD118	Endoscopic aspiration of cerebellar cysts and insertion of Ommaya reservoir	12 months	Resolution of hydrocephalus and signs of raised ICP. Reduction in cyst size on neuroimaging.
Taglia et al. 2018 [25]	23	Male	Bilateral pyramidal syndrome and left arm dystonic posture	Seizures from 2 months of age.	MRI & CT calcifications in basal ganglia, subcortical white matters of both hemispheres and right dentate nucleus	Two heterozygous mutations: n.57A>G and n.*10G>T	N/A	N/A	The man’s 19-year-old brother had a similar clinical and radiological picture and has the same result of genetic tests.

LCC may present with acute symptoms. The commonest presenting features are pyramidal signs, seizures and a progressive cognitive deficit. The cysts exert mass effect and are directly related to the neurological manifestations of the disease [24]. LCC is restricted to the central nervous system; extra-neurologic manifestations have only been described in three patients, with two having café au lait spots and a third demonstrating cysts in the liver, kidney and pancreas [6, 29]. A related condition, Coats syndrome, is also caused by a microangiopathy; in addition to the cerebral changes, patients with Coats syndrome also have retinal angiopathy, causing bleeding and exudative retinopathy [29]. Coats syndrome is caused by a mutation in the CTC1 gene, located upstream of the SNORD118 gene on chromosome 17p; the mutation affects telomere maintenance and is unrelated to the SNORD 118 mutation in LCC [3]. The Coats Plus syndrome, also related to anomalies in telomere maintenance, has similar neurological manifestations, but is associated with systemic features, including premature greying of hair, anaemia and osteoporosis.

The diagnosis is made primarily on the characteristic triad on radiology. Infections, such as tuberculosis and cytomegalovirus need to be excluded. Fahr disease, Sturge-Weber syndrome and MELAS also demonstrate calcifications, but do not have cysts or leukoencephalopathy [29]. Astrocytoma is an important differential. . However, magnetic resonance spectroscopy (MRS) shows a low choline level in the involved brain, consistent with the high water content in the parenchyma [29]. MRS also shows reduced NAA levels, and no metabolites are evident in cyst fluid. Astrocytomas, by contrast, demonstrate a high choline peak, consistent with rapid membrane turnover. As a biopsy was undertaken in our patient, MRS was not required. Other potential differential diagnoses include Alexander’s disease, where cysts and white matter changes are not associated with calcification. In this context, therefore, most cases of LCC can be diagnosed without biopsy or genetic studies.

Few children have been followed up for a long time; in the published paediatric cases we identified, the mean follow-up is 13 months. Natural progression of the disease is not well described. The microangiopathy persists, leading to the development of new cysts or the growth of existing ones within areas of leucoencephalopathy. An adult patient who presented at 44 years and was followed up for 8 years required multiple craniotomies to excise recurring frontal and cerebellar cysts [7]. One patient only received mannitol to control mass effect from the cyst, with subsequent reduction in its size [29]. Many reported cases focus on those who have developed symptoms in adulthood. These symptoms may present suddenly or at a slow, insidious rate. A 69-year-old woman began to show symptoms at age 37 and demonstrated diffuse bilateral leukoencephalopathy on MRI [6]. A gradual decline in both cognition and motor ability occurred over the next 32 years [6]. In another patient, MRI scans at the age of six showed bilateral cysts in the cerebral hemispheres. It was only at 18 years of age that imaging showed the typical LCC triad, demonstrating the progressive nature of LCC, underlining the importance of long-term follow-up [9].

The management of LCC in children is particularly challenging. In some cases, early presentation portends a more aggressive clinical course (Ref Jenkinson 2016). Many of the patients described in Table 1 underwent excision of cysts from the frontal lobe or cerebellum, but as many cysts are located in the thalamus or basal ganglia, this is often not possible without incurring significant neurological deficit. In the context of a progressive neurological disease, that is unpredictable and can also remain stable for a long time, minimally invasive surgical options, such as cyst drainage and insertion of an Ommaya reservoir, are preferable. One study describes the use of biweekly injections of VEGF inhibitor Bevacizumab for one year; this led to a reduction in cyst volume and white matter changes, with a corresponding reduction in bradykinesia, with a plateau at 6 months (ref Fay, 2017).

## Conclusion

Labrune syndrome, also known as LCC, is a rare ribosomopathy with clear diagnostic criteria. Management in children is aimed at reducing mass effect by draining cysts. In the presence of a clear radiological triad, biopsy and genetic studies are not necessary. As the disease process is progressive, long-term follow up is critical. A higher awareness of this rare disease among paediatric neurosurgeons is critical.
